# Approaches used by parents to keep their children safe at home: a qualitative study to explore the perspectives of parents with children aged under five years

**DOI:** 10.1186/s12889-015-2252-x

**Published:** 2015-09-29

**Authors:** Joanne Ablewhite, Lisa McDaid, Adrian Hawkins, Isabel Peel, Trudy Goodenough, Toity Deave, Jane Stewart, Michael Watson, Denise Kendrick

**Affiliations:** Division of Primary Care, University of Nottingham, Nottingham, NG7 2RD UK; Clinical Research & Trials Unit, Norfolk and Norwich University Hospital NHS Foundation Trust, Norwich, NR4 7UY UK; Emergency Department, Nottingham University Hospitals NHS Trust, Nottingham, NG7 2UH UK; Faculty of Health and Applied Sciences, University of the West of England, Bristol, BS16 1QY UK; School of Health Sciences, University of Nottingham, Nottingham, NG7 2HA UK

**Keywords:** Child safety, Child injury prevention, Safety strategies, Qualitative

## Abstract

**Background:**

Childhood unintentional injury represents an important global health problem. Many unintentional injuries experienced by children aged under 5 years occur within the home and are preventable. The aim of this study was to explore the approaches used by parents of children under five in order to help prevent unintentional injuries in the home and the factors which influence their use. Understanding how parents approach risk-management in the home has important implications for injury practitioners.

**Methods:**

A multi-centre qualitative study using semi-structured interviews. A thematic approach was used to analyse the data. Sixty five parents of children aged under 5 years, from four study areas were interviewed: Bristol, Newcastle, Norwich and Nottingham.

**Results:**

Three main injury prevention strategies used by parents were: a) Environmental such as removal of hazards, and use of safety equipment; b) parental supervision; and c) teaching, for example, teaching children about safety and use of rules and routine. Strategies were often used in combination due to their individual limitations. Parental assessment of injury risk, use of strategy and perceived effectiveness were fluid processes dependent on a child’s character, developmental age and the prior experiences of both parent and child. Some parents were more proactive in their approach to home safety while others only reacted if their child demonstrated an interest in a particular object or activity perceived as being an injury risk.

**Conclusion:**

Parents’ injury prevention practices encompass a range of strategies that are fluid in line with the child’s age and stage of development; however, parents report that they still find it challenging to decide which strategy to use and when.

## Background

Childhood unintentional injury represents an important global health problem [1]. In England and Wales, unintentional injury is the second leading cause of mortality and a significant cause of morbidity within the 0–4 year age group [2]. In addition to deaths, many non-fatal injuries occur each year which may result in hospitalisation or visits to primary care services. In 2002, the latest year for which detailed emergency department (ED) data is available, unintentional injury accounted for 416,806 ED attendances in children aged under 5 years [3]. Most unintentional injuries to children under 5 years occur in and around the home as this is where they spend the majority of their time [4].

Birth to 5 years old is a time of rapid changes in children’s physical and cognitive abilities which can increase their risk of unintentional injury [5]. Changes in the child’s developing mobility, cognitive ability and receptive understanding, require different parental anticipatory and safety strategies in order to minimise potential injury risks. Evidence suggests three risk-management strategies that parents of young children routinely use to reduce injuries within the home: Environmental changes to prevent access to hazards, supervision and child based strategies such as teaching about safety [6]. In reality parents use these preventative strategies in combination [7].

Environmental strategies include for example, having a working smoke alarm, the safe storage of hazardous objects and the installation of safety equipment.

Parental supervisory approaches are also important. However, there is no universal definition of supervision that is used by parents, researchers and practitioners [8]. Parental supervision encompasses a spectrum of visual and listening strategies in combination with the proximity of the child to the parent should it be necessary for them to intervene [8]. Parental supervision can reduce young children’s risk of unintentional injury within the home [7, 9–12]. Parental perceptions that supervision is effective in reducing a child’s risk of injury at home are also important [13–15]. A range of factors may influence the ways that parents supervise their children, for example, by child gender [9, 16]. In addition, research has found child character also shapes parental supervision [17, 18].

Teaching strategies for reducing, explanations, example and rule, and approaches to modifying child behaviour, such as rewards and punishments [19, 20]. Teaching strategy may vary with parenting style; for example, permissive parenting has been associated with greater explanation and less rule usage [19]. Others have found differences in the way that parents teach rules according to age: parents of younger children (aged 2–2.5 years) were found to repeat rules, whereas parents of older children (aged 3–3.5 years) began to explain the reason for the rule [21].

In addition to evidence relating to parental injury prevention approaches, theories of behaviour change and theoretical models can facilitate understanding of approaches to injury prevention. Numerous theoretical models have evolved that aim to predict, explain and potentially alter decisions related to health behaviours. Some focus on individual behaviour change such as the Health Belief Model or Social Cognitive Theory [22]. Individual behaviour change theories provide a conceptual basis for the development and evaluation of injury prevention interventions. However, individual behaviour change models must be considered in the broader context of an ecological approach. An ecological approach to injury prevention involves consideration of the many factors and influences that may impact on parental approaches to injury prevention within the home [23].

In this paper we discuss findings from a qualitative multi-centre study. The aim of this study was to explore the perspectives of parents of children aged under 5 years, with regard to the approaches they use to prevent unintentional injuries at home as a part of their everyday lives. Understanding how parents approach injury prevention within the home in the home has important implications for injury practitioners.

Findings of this study regarding the barriers and facilitators to preventing unintentional injury within the home are reported elsewhere [22].

Approvals for the study were granted from the North Nottinghamshire Research Ethics Committee (Ref: 09/H0407/14) and the Research and Development Offices in each of the NHS Trusts where recruitment took place.

## Methods

### Recruitment and selection

This interview study was a nested study undertaken alongside multi-centre case–control studies exploring risk factors for falls, poisoning and scalds which are described elsewhere [24–26]. A total of 65 participants were recruited from four study centres: Nottingham, Bristol, Newcastle-upon-Tyne and Norwich. Maximum variation sampling [27] was used to ensure participants covered a range of child ages, gender, injury type and deprivation of area of residence using the rank of multiple index of deprivation [28] (see Table 1). As there were low numbers of parents with children with scald injuries in Norwich, additional participants were recruited at two study centres (Bristol and Newcastle).

**Table 1:** Demographic characteristics of the sample

	Fall	Scald	Poisoning	Control^a^	Total
Child age					
Under 1	2	0	4	0	6
1	5	4	7	4	20
2	4	7	4	7	22
3	4	4	1	3	12
4	1	1	0	2	4
Gender					
Male	9	10	8	10	37
Female	7	6	8	6	27
Deprivation					
> = median	6	8	7	8	29
< median	10	8	9	8	35
Accommodation					
Rented	7	11	4	2	
Own home	8	6	12	14	
Other	1	0	0	0	
Number of children <16					
1	8	8	7	7	30
2	4	5	4	5	18
3	2	1	3	3	9
4	1	2	2	0	5
5	0	1	0	1	2
6	1	0	0	0	1

^a^Cases included children under-5 attending A&E services or admitted to hospital with a fall, suspected poisoning or poisoning or scald injury that happened at home. Controls included children under-5 from the same or a neighbouring GP practice as the case who had not attended an ED or been admitted to hospital on the day the case child attended ED or was admitted to hospital

Participants that were recruited to the case–control studies face-to-face were given information during recruitment about three nested studies, which included this study, and asked if they would like to take part in one. Participants who were recruited by post, who indicated an interest in taking part in a nested study, were contacted by telephone by a researcher. The researcher explained the studies to them and asked if they would like to take part in one. Parents that became a participant in either of the two other nested studies were excluded from taking part in this study. Participants were reimbursed with a £5 shopping voucher as an inconvenience allowance.

### Data collection

An interview guide was designed based on findings from the systematic reviews of barriers and facilitators to injury prevention [17, 29] to explore parental perceptions of injury prevention strategies, child home injury risks, and factors that might help to prevent child injuries as well as descriptions of injury events and near misses. Four pilot interviews were undertaken; in two sites resulting in minor word changes and additional prompts. Pilot data were not included in the analysis. This paper reports on the strategies used by parents to reduce child injuries in the home. The findings from other topics covered in the interview will be reported elsewhere [30]. Semi-structured interviews took place in participants’ homes and lasted an average of 40 min. Interviews were conducted by experienced researchers based in each of the study centres. Interviews were conducted between March 2011 and July 2012. Written informed consent was obtained from participants prior to commencing the interview.

### Data analysis

The interviews were audio recorded and transcribed verbatim, data were anonymised prior to transcription. Data were analysed using a thematic approach: Initially data were read and re-read drawing out emerging themes and sub-themes; a coding structure was developed by randomly selecting four transcripts that were read by an independent research consultant, a lay research advisor and two researchers from different study sites. As the coding structure was applied to subsequent interview transcripts, other themes that emerged were discussed and agreed until a final coding structure was applied to all interview data. Analysis was facilitated using the software Nvivo (version 9). Throughout this process regular discussions were held with the research team to review emergent findings and ensure consistency of approach.

## Results

Sixty five parents of children aged less than 5 years took part in an interview; 49 parents, whose child attended a hospital emergency department with a fall (16), scald (17) or poisoning (16). Controls included children under-5 from the same or a neighbouring GP practice as the case who had not attended an ED or been admitted to hospital on the day the case child attended ED or was admitted to hospital. The participant characteristics are shown in Table 1. All interviews were conducted with mothers; fathers were also present in three interviews. One interview with a parent whose child had a scald was excluded from analysis due to an inaudible interview recording.

### Parental approaches to injury prevention within the home

Parents described three broad strategies to help prevent child injuries in the home: Environmental, parental and teaching. The findings indicate that similar injury prevention strategies were used by parents of children who had experienced a fall, scald, or poisoning injuries and those of children in the control group. It can be seen that environmental modifications were the most common strategy used to reduce injury risk; in comparison, fewer parents used educational strategies. Quotes to support the themes are shown in Table 2.

**Table 2:** Representative quotes

Theme	Sub theme and representative quotes
Environmental strategies	Out of reach and restricting access
	*Yeah and any medicines… ‘cause at the moment we have all got colds and coughs again they are on top of the… kitchen bench pushed to the back but again they all have the child locks on. They are not in a cupboard because we are actually using them regularly but normally all the medicines are at a height that even I have trouble to reach so they wouldn’t be able to reach those at all*. (Mother of girl age 4, Control, <median IMD).
	*What we try to do all the time is to ensure that we don’t put things that she is able to reach. She is a bit ‘touchy’ so we try to keep away all things that we perceive to be dangerous… We try to keep these chairs away from her. We try not to put things on top of the desk for her to be able to reach. We keep bottles, medicines, cooking utensils, irons and almost everything she is likely to touch so we just try to leave her toys on the floor for her play mostly….she is very, very active* (Mother of girl age 2, Scald injury, >median IMD).
	*There’s two doors that we always keep closed, which is the bathroom door and the erm, kitchen door,– because those are the two rooms that we can’t really make safe… Erm, and the same with the kitchen, we only let–allow them to go in the kitchen when there’s an adult with them*. (Mother of boy age 3, Poisoning injury, < median IMD).
	*Yeah I have just discovered that I need to erm to keep my door locked because [Child Aged 2] can reach that handle now. About two weeks ago he couldn’t so you know they just surprise you all the time don’t they with new things* (Mother of boy age 2, Control, < median IMD).
	*We try and keep all kind of small parts of toys out of reach which is harder now she is getting taller and taller. Erm things like keeping knives and that out of her reach again now because she is moving chairs up to things that is getting quite a tricky*. (Mother of girl, age 1, Control, >median IMD).
	Tidy home
	*But there are ways like I said you can try and prevent them I always like to keep this floor tidy. Nothing on the stairs, you know, so they can’t fall over going up the stairs and… I do generally keep the floor tidy. I think that would help because they can’t fall over anything then can they*? (Mother of boy age 3, Control, <median IMD).
	Adapting the home
	Water temperature
	*My water is set at a certain degrees so it doesn’t come through hot enough for him to burn himself*. (Mother of boy age 2, Fall injury, < median IMD).
	Rooms perceived as having greater risks of injury
	*Sharp knives and anything like that they are all kept up but I have got a bungee rope did you notice it in the kitchen? From the top cupboard on to the cooker because he would pull the cooker down and stand on it and climb on the work surfaces. Right the cooker rocks I have had duct tape I have had this industrial tape to keep the bottom oven door shut none of it worked until we put the bungee rope on and he didn’t touch it*. (Mother of boy age 2, <median IMD).
	Accommodation factors
	*It varies from landlord to landlord …The only thing about living here is the fact that there is no carpet downstairs. Erm we have always had carpet and when we moved here I was just a bit worried about him falling on this floor obviously coz it’s although wood is soft it’s not as soft as when you have got carpet on… I mean he fell down like hence why he ended up in hospital*. (Mother of boy age 2, Fall injury, <median IMD).
	Safety equipment
	*Erm, I’ve got all the plug sockets in, I have got the stair gates, erm, we’ve gone to the extent of radiator covers now because they were touching the radiators while they were on and obviously burning their fingers a little bit.. I’ve tried fridge locks, I’ve tried freezer locks–I will literally try anything and everything that’s about* (Mother of boy age 1, Poisoning injury, >median IMD).
	*…I still keep the sockets covered now just in case. That’s the only thing really because nothing worse than a baby crawling towards a socket.* (Mother of boy age 3 Control, >median IMD).
	*I don’t have any cupboard locks because I found that the children* [are] *actually more inquisitive if they can’t get in to it. So I actually let them lose in things like the saucepan bits and… they went through a stage of banging doors and I just know that the more you try to stop them, the more they did it. We let them do it and then in a day it was done*. (Mother of girl, age 3, Poisoning injury, > = median IMD).
	Safety equipment and the ages and stages of child development
	*I did have stair gates but I took them off because they can climb over them… So yeah, I thought it was safer to remove them rather than leave them there*. (Mother of girl age 2, Poisoning injury, > median IMD).
	Safety equipment reactive and proactive approaches
	*we haven’t really put locks and barriers up, trying to, we may put a lock on the kitchen cupboard with the cleaning things in it if [F] is showing an interest in cupboards and he’s not showing an interest in following rules, but it worked well with the first so and then as they get older, you know, it’s just about teaching them… but if you can’t just assume that they’re going to learn. So we may have to resort to barriers for certain areas and that’s fine, you know, we’ll do that if necessary.* (Mother of boy age 3, Scald injury, <median IMD).
Parental strategies	Parental supervision
	*I think the most predominant thing is I rarely… I just don’t leave them on their own whilst she is so small. She is only what 20 months now, so I tend to just have to watch her all the time…*(Mother of girl age 1, Fall injury, <median IMD,)
	*I think you just have to do the best like keep an eye on them when you can but obviously you can’t always… If he goes quiet I just have to make sure he is not doing anything bad [laughing].* (Mother of boy age 2, Control < median IMD).
	Parental supervision interrupted by distractions/household tasks
	*Just that it’s really hard you know it’s really hard especially when you are on your own you know if you have got a mother and father, fair enough, it’s a bit more easier but like I say I mean I said it before it’s a bit of a hassle to keep taking [Child’s Name] everywhere it’s not because I know she is safe then but it would be a lot easier if I had like a dad here just to say well I will keep an eye on her I will just go upstairs or I’ll go do the ironing*. (Mother of girl age 1, Fall injury, female, <median IMD).
	*I mean if I’ve got household task to do I kind of run between the kitchen and here in the lounge just to check on him and make sure he’s not doing anything crazy, but most of the time I don’t like to do that because I find it really stressful. I only do that if I really have to. You know if I really need to make some lunch so that we can eat*. (Mother of boy age 1, Poisoning injury, <median IMD).
	*It’s basically just keeping your eye on them. We don’t leave her for too long and I know it only takes seconds we do try and make sure that if we are not watching her that our 7 year old is aware that she is on her own as we do tell him. The idea is that he we are not leaving him in charge of her we are asking him to just we are often saying to him what is she doing what’s she doing and he gets really cross and says she’s fine.* (Mother of girl age 1, Control, > median IMD).
	*Erm now she is moving when she was younger and she was just sitting somewhere she was there and you could keep an eye on her but now she is like in here out here she wants to be kept busy all the time*… (Mother of girl age 4, Fall injury, female, <median IMD).
	Parents modify their own behaviour
	*sometimes what I do is erm just not doing things in front of her to avoid her thinking about it. Like I never plug and unplug things in front of her. I just wait for her to turn around and I use the socket without her [seeing] so she wouldn’t… obviously they are going to do what you are doing so I tend to avoid her to see me*. (Mother of girl age 1, Fall injury, >median IMD).
Teaching strategies	Learning about hazards
	*So for instance she is much better since she tried to get into the oven and I have been telling her you can’t open the over door…we actually had to go over and show and put her hand very close and say this is heat this is hot, this is last night’s conversation, if you do this it is going to hurt, which is far better if she has actually experienced it then trying to explain it to her*. (Mother of girl age 3, Fall injury,>median IMD).
	*Erm we tell them not to run in the house they have a star chart with behaviour and running is one of the key erm points. They run they get a black cross erm he has been [Child’s Name] in particular who is very energetic he has been told and taught how to come down the stairs nicely the top of the stairs are very narrow and the stairs are steep and so ever since he started to crawl he has been watching and learning how we come down the stairs and now he can actually walk down the stairs holding on to the banister and he knows erm that it is steep and he has to be careful erm mainly through watching watching [Older Child’s Name] and my husband and I how we act*. (Mother of boy age 3 > median IMD).
	*They have got to learn …I think age is important too because obviously you can’t show a child who is obviously not going to understand what you are showing it, or isn’t aware of what is actually going on. As they get older you have to obviously you have to explain in depth as to why things are dangerous and why you can’t do that so they know…*(Mother of boy age 1, Fall injury, <median IMD).
	Controlled risk taking
	*As I say teaching as early as you can and sometimes they can have these little accidents so they learn that if you do that it will hurt cos these two use to jump off the end of the sofa and once I let them do it and think he hurt his knee and he hasn’t done it since*. (Mother of boy age 1, Scald injury > median IMD).
	*I think it’s educating them as to why we do it because you can just say no no so many times unless the child knows why you are saying no I don’t think it goes in. It is not that all the time they have to experience it themselves but there has to become that understanding for them to want to do it*. (Mother of girl age 1 Control, > median IMD).
	*I mean I said I don’t use everything, because I think you know your children and I think equally it’s, I think you can have overkill on locks and things like that. I think that couldn’t, in the long run, I’m not sure it particularly helps, I don’t know, that’s from my experience. I think it just makes children, you know, what have you got locked in that cupboard… I didn’t, to be fair I just didn’t want loads of locks over the cupboards, because I just felt that it would cause more problems long term* (Mother of girl age 3, Poisoning injury, >median IMD).
	Near miss as a deterrent
	*We haven’t done any plugs or any corner things we haven’t done any safety measures erm and again it’s about teaching him and to be fair at the moment he hasn’t really shown any interest in plugs and sockets that’s been an advantage but you know if he is doing anything that we think is dangerous then we kind of teach him say no and give him something else to do erm he has probably learnt a bit the hard way you know if he has banged himself then actually he is careful the next time*. (Mother of boy age 1, Control, <median IMD).
Most important strategies	Using a combination of strategies
	*Supervising them, being aware of what they are doing. Being aware that they can change within a week. I think that as well as teaching them and reinforcing it* (Mother of boy age 2, > median IMD).
	*To me and that’s it’s educating her constantly supervising to make sure she is actually sticking to what you tried to teach her*. (Mother of boy age 1, Control, <median IMD).
	Uncertainty over which strategy to use in line with ages and stages of child development
	*You look forward to the next stage and you’re all ‘what are they going to do next, what are they going to learn next?’ and you kind of assume that it’s going to be a long time… Yeah, so I think you’re just thinking it’s going to be a long time. Like with walking he started walking a little bit and I was like–I was writing my diary ‘oh, he’s walking a little bit, it will be a month or so before he starts walking I think’ and it was a few days… you see the beginnings of it and think ‘oh, I’ve got a bit of time to make arrangements’ you know if you need to do something like with a cupboard and–no actually it surprises you how quickly they change* (Mother of boy age 1, Poisoning injury, < median IMD).

### Environmental strategies

Creating a safe environment for children was an important safety strategy for parents within the home. This included keeping dangerous objects out of reach (e.g. hot drinks, sharp implements, glass, and poisonous substances), restricting access to certain areas, locking windows and external doors, keeping the water thermostat on a low temperature and ensuring the home is kept tidy to avoid trip hazards. The majority of parents used some form of safety equipment to help create a safe environment for their child. The types of properties parents lived in varied widely in terms of their size, ownership and age with each providing distinct issues to make the home environment a safer place for children. Typically, the kitchen and bathroom were considered to be the rooms with more injury hazards and a greater risk of injury.

#### Out of reach and restricting access

Parents described how they put dangerous objects up high or to the back of work surfaces to prevent children from reaching them. Some parents thought it was important to store items in a safe but accessible place otherwise they may not be returned after use. For example, one parent said that because the cold and flu medications were being used regularly at the time of the interview, these were not kept in their usual storage place. Some parents described how medicine such as Calpol may not be stored out of a child’s reach because it has a cap they perceived as child resistant. (Calpol is a brand of children’s medicine the main ingredient is paracetamol suspension).

A particular focus for many parents was having strategies for the bathroom and kitchen or restricting access to these rooms, as they were generally considered to be the places where accidents are most likely to occur. Keeping objects out of reach was said to become more difficult as children grow older.

#### Adapting the home

Parents described a range of ways in which they adapted their home in order to make it a safer place for children. One of these was keeping the home tidy and free from clutter to reduce trip hazards. However, for some parents this was made more difficult due to a lack of space or appropriate storage in the home. Another approach was to turn the water temperature on the boiler thermostat down to a level which parents believed would not burn a child should they come in contact with it. Some parents described ensuring that children were unable to escape or fall from windows or external doors by locking these. Often parents were taken by surprise at how suddenly these areas become accessible as the child becomes mobile.

#### Accommodation factors

Accommodation factors were described by parents as impacting on their approaches to keeping their children safe at home. Living in temporary, rented and social housing was described as a challenge by some parents as they were unable to adapt their home environment as they would like. Other parents stated that a lack of safe indoor or outdoor play space made it difficult to make the home environment safe for children. Older homes were considered to have a number of safety issues as they have not been built to the same modern standard as new homes.

#### Safety equipment

Parents used a range of equipment to help create a safe environment for their children. The most common types of equipment used included stair gates, plug socket covers, furniture corner protectors and safety locks or catches. Other forms of safety equipment less frequently mentioned included blind cord cleats, cot bars, smoke alarms and fire guards. Safety equipment was not thought to be infallible and did not replace the need for other injury prevention approaches, in contrast to some parent accounts of child resistant caps.

The need for and effectiveness of a number of safety devices typically reduced with child age and development and this required parents to reassess their use. For example, parents of post-toddler age children in the study talked about how their child would start to climb over the stair gate or learn how to open it. This would then leave them with the dilemma of whether it was safer to keep it in place or remove it as the risk of harm to the child from climbing was seen as greater than not having the gate there at all. The limitations of other safety equipment were described by some parents in terms of children being able to pull off socket covers, remove child resistant caps from medicine bottles and open child door safety locks. Some parents believed that safety devices could be a hindrance, as inquisitive children are likely to be drawn to what they cannot access.

Some parents were reluctant to create an ‘artificially’ safe environment in their own home as this would not be the same in other people’s homes or different environments, so they relied on teaching strategies instead.

Approaches to using safety equipment in the home were described by parents as either reactive (e.g. they do not have cupboard locks if the child has not displayed an interest in the cupboards) or proactive (e.g. installing safety equipment just in case).

### Parental strategies

Parental strategies included supervising children in the home and parents modifying their own behaviour in order to reduce injury risks.

#### Parental supervision

Parents used various strategies to supervise their children, from those who claimed to ‘*watch their children like a hawk*’ (constant visual and audio supervision) to those who ‘*keep an eye on them*’ and listen for silence as a cue for checking (intermittent visual and audio supervision). Supervisory practices varied by child age; parents of babies and children under 12 months described more direct and constant supervision.

Parents talked about how their ability to supervise children can be impaired by daily distractions and trying to get on with household tasks, such as cooking and cleaning. This was particularly the case for lone parents, parents with two or more children and two-worker households, although in some cases parents with older children used them as extra pairs of eyes to let them know if a younger one was engaging in a potentially unsafe activity. Some parents talked about the difficulty of supervising children and juggling other competing tasks.

Parents spoke about the difficulties of supervising children as they develop and become more active as they grow. Parents talked about balancing between keeping their children safe as they become increasingly capable and mobile and giving them the freedom to learn and explore.

#### Parents modify their own behaviour

Another strategy adopted by parents was to modify their own behaviour in order to minimise injury risk to children. This involved doing activities and household tasks which they considered to be dangerous when their children were not around. By avoiding these behaviours in front of children, parents hoped this would also prevent replication of behaviour.

### Teaching

Teaching was described in terms of children learning about hazards, giving children basic safety advice and rules, allowing children to experience things through controlled risk and parents leading through example. Teaching was considered most effective when children were old enough to understand personal safety, though parents found it was difficult to put an age as to when best to begin as children develop at different rates. Parents suggested that the main advantage of children learning about safety is that they then can apply this to different environments.

Parents described that there was an element of trust when it came to teaching children and whether parents felt that they will follow rules when left unsupervised. Some parents thought it important that children are exposed to controlled risks in order to learn from experience. Near misses or old injuries could often work as a deterrent against future risk taking. Parents described that when a child is tired or in a bad mood then they may not be receptive to teaching or following safety rules.

### Most important strategy

Parents were asked to indicate which injury prevention strategy they thought was *most* important. Parents often found it difficult to narrow this down to one approach and the child’s age and stage of development were central to such considerations. Using a combination of the three main strategies was often seen as the best way to prevent injuries in the home, as each was thought to have its limitations. Importantly, each child differs in their development stage and character so parents adapted their injury prevention strategies accordingly. Those with very young children (aged 1 year and under) were more likely to rely on supervision and safety equipment. This typically shifted to a focus on education as they got older, which was thought to be more beneficial as it can be applied to different environments. Parents felt they could trust some children more than others, for reasons such as their character or whether the parent thought the child had an adequate grasp of the safety risks; however, a key factor in this was increased age. A concern raised by parents during the interviews was that it was difficult to know which strategy to use according to child age and development.

Supervision has an essential role to play in keeping children safe according to the parents interviewed, especially those with young children. However, parents acknowledged that it is not possible to supervise all of the time and so strategies are needed to compensate for when they cannot supervise as well as they would like. Safety equipment proved slightly more contentious. Amongst those parents who considered this strategy as most important, stair gates were frequently cited as the most useful safety product to prevent child injuries. However, some parents felt that safety equipment alone was not sufficient to keep children safe, while others held the idea that you can ‘*overkill*’ with equipment and allowing a certain amount of risk enables children to develop the skills they will need to negotiate safety issues themselves as they get older. Indeed, when talking about the most important injury prevention strategies, parents frequently referred to using common sense and being mindful of potential risks.

Teaching children about safety and what is and is not dangerous was cited as the most important strategy. The advantage was the transferability of the strategy to different environments. However, this strategy was considered to be less effective with young children as they are unable to understand and there was debate over when is the right time to start teaching children safety rules. Most parents thought that teaching should begin when children were capable of understanding and following safety rules, though others suggested that this process should start from infancy.

## Discussion

This paper presents key findings of a multi-centre qualitative study that explored parental approaches to keeping their children safe from unintentional injury at home. Our findings add to the paucity of qualitative literature on parental accounts of their approaches to unintentional injury prevention within the home. The findings are important as there is little research, conducted in the UK, and that took place in family homes, to find out what approaches are being used as a part of everyday lives.

The study found that parental approaches focused on three main injury prevention strategies: environmental, parental supervision and teaching children about safety and using safety rules and routines. The study found that parents describe using a combination of these In addition, the use of strategy and perceived effectiveness were fluid processes and depended on child age and character, stage of development and previous or near miss injury experiences. Importantly, the study found that some parents described being unsure about which strategy to use and when.

### Strengths and limitations

The maximum variation sampling strategy ensured the perspectives of a diverse range of parents were included, as a multi-centre study this allowed flexibility, for example, when in one study centre there were low numbers of children with thermal injuries it was possible to recruit additional participants via two other study centres. Generalisations from this study should be made with caution however; the maximum variation sampling and the large number of interviews conducted helped to obtain a wide representation of parental accounts. These should be broadly transferrable to parents of young children living in similar situations.

However, the study has some limitations. In addition, parents participating in the interviews may have been those most interested in child safety and their experiences may not reflect those of other parents. Social desirability bias [31] may also have occurred with parents wishing to present themselves as “good” parents and being reluctant to reveal if they did not undertake certain safety practices.

In emphasising the importance of context and the multiple influences on the approach to home safety for the under 5’s taken by parents this study reflects findings from other studies [7, 14]. Consistent with other studies parents recognised that parental supervision has a key role in keeping children safe from injury. Some parents described using listening as a supervision strategy but this is known to have the potential for an increased risk for injury, particularly for boys living in circumstances of socio-economic deprivation [15].

The parents in our study reported using a range of environmental strategies, particularly in the kitchen and bathroom, known to prevent poisoning and thermal injuries. However, parents that lived in rented accommodation described this as limiting the environmental strategies they could use, particularly with regard to installing safety equipment. Whilst some parents seemed to over-rely on safety features such as child resistant caps for medicine bottles, others were more sceptical about the value of environmental strategies.

Teaching children about safety and what is and is not dangerous was cited as the most important strategy; it has the advantage of being transferrable to different environment. Many parents reported using teaching, but as some of the quotes demonstrate parents were using this strategy for even the youngest children. Parents reported trusting that their child would follow rules when left unsupervised and some felt children should be exposed to controlled risks to enhance their learning. However, teaching, if not used in conjunction with other strategies has the potential to increase the risk of injury [21]. Our study demonstrated that most parents thought that teaching should begin when the child was capable of understanding the safety rules. However, it has been shown that teaching as the primary injury prevention strategy is only valid when a child has a high level of understanding about the safety issue, and that teaching in ways that promote the acquisition of a thorough understanding of the safety issue and not just an awareness of a safety rule is essential if teaching as a risk management strategy is to reduce young children’s injury risk [21].

Parents demonstrated concern that it was difficult to know which strategy to use according to the child’s age and development and they recognise other factors come into play such as the child’s temperament, and how a child may be feeling at a given point in the day. Whilst some parents anticipate developmental changes and introduce safety equipment ‘just in case’ others have a more reactive approach and do not consider installing safety equipment until the child displays interest in a potential risky situation. However, many parents were surprised at the speed of development and how quickly areas that one day proved no risk could very quickly become accessible as the child becomes mobile.

Alongside this parents face a variety of challenges in their quest to keep their children safe from unintentional injury–the ability to supervise can be more difficult for lone parents, and the ability to make environmental changes more difficult or even not possible for those living in rented accommodation.

An ecological approach that takes account of the complexity of factors that shape parental injury prevention strategies is important. Developing understanding of such influences and how these relate to the fluid nature of how parents keep their children safe from injury within the home is crucial.

### Implications for research and practice

This study highlights that whilst there are certain known risk factors for particular kinds of unintentional injury for young children at a population level, just being aware of this may not necessarily support the parent in helping keep their child safe in the particular set of circumstances in which they live their lives and parent their children. Those that work with parents need to understand the parent’s beliefs and current attitude to child safety in the home. They need to avoid offering advice that cannot be implemented and to help the parent think about which strategies they could use that offer the best fit to minimising child injury risk in the given circumstances. They need to help parents anticipate next steps they may need to take.

In terms of future research, consideration should be given to the possibility of developing some kinds of decision aids that could help parents and practitioners be jointly and actively involved in considering what is known about child injury prevention and what injury prevention strategies might be best to apply given parents’ own set of personal circumstances.

## Conclusion

In conclusion, this study has identified the key safety strategies used by parents in the home to reduce unintentional injuries. Parents’ injury prevention practices encompass a range of strategies that are fluid in line with the child’s age and stage of development; however, parents report that they still find it challenging to decide which strategy to use and when.

## References

[1] Peden M (2008). World report on child injury prevention.

[2] ONS (2011). Table 2 deaths registration summary statistics. England and Wales 2011.

[3] Department of Trade and Industry (1999). Home Accident Surveillance System including leisure activities 21st Annual Report 1997 Data. Accident data and safety research. Home, garden and leisure. Research commissioned by the Government’s Consumer Affairs Directorate at the Department of Trade and Industry.

[4] Audit Commission (2007). Better safe than sorry. Preventing unintentional injury to children. National Report (Health).

[5] Sethi D, Towner E, Vincenten J (2008). European report on child injury prevention.

[6] Morrongiello BA, Ondejko L, Littlejohn A (2004). Understanding toddlers’ in-home injuries: I. Context, correlates, and determinants. J Pediatr Psychol.

[7] Morrongiello BA, Ondejko L, Littlejohn A (2004). Understanding toddlers’ in-home injuries: II. Examining parental strategies, and their efficacy, for managing child injury risk. J Pediatr Psychol.

[8] Morrongiello BA (2005). Caregiver supervision and child-injury risk: I. Issues in defining and measuring supervision; II. Findings and directions for future research. J Pediatr Psychol.

[9] Morrongiello BA, Dawber T (1998). Toddlers’ and mothers’ behaviors in an injury-risk situation: implications for sex differences in childhood injuries. J Appl Dev Psychol.

[10] Morrongiello BA, Kiriakou S (2004). Mothers’ home-safety practices for preventing six types of childhood injuries: what do they do, and why?. J Pediatr Psychol.

[11] Schwebel DC, Bounds ML (2003). The role of parents and temperament on children’s estimation of physical ability: links to unintentional injury prevention. J Pediatr Psychol.

[12] Morrongiello BA, Corbett M, McCourt M, Johnston N (2006). Understanding unintentional injury-risk in young children I. The nature and scope of caregiver supervision of children at home. J Pediatr Psychol.

[13] Sparks G, Craven MA, Worth C (1994). Understanding differences between high and low childhood accident rate areas: the importance of qualitative data. J Public Health Med.

[14] Garling A, Garling T (1995). Mothers’ anticipation and prevention of unintentional injury to young children in the home. J Pediatr Psychol.

[15] Ablewhite JK, Kendrick D, Watson M, Shaw I. Maternal perceptions of supervision in pre-school-aged children: a qualitative approach to understanding differences between families living in affluent and disadvantaged areas. Prim Health Care Res Dev. 2014;16(4):1–10. 10.1017/S1463423614000218 24871079

[16] Morrongiello BA, Hogg K (2004). Mothers’ reactions to children misbehaving in ways that can lead to injury: implications for gender differences in children’s risk taking and injuries. Sex Roles.

[17] Ingram JC, Deave T, Towner E, Errington G, Kay B, Kendrick D (2012). Identifying facilitators and barriers for home injury prevention interventions for pre-school children: a systematic review of the quantitative literature. Health Educ Res.

[18] Ablewhite J, Kendrick D, Watson M, Shaw I (2014). Maternal perceptions of supervision in pre-school-aged children: a qualitative approach to understanding differences between families living in affluent and disadvantaged areas. Prim Health Care Res Dev.

[19] Morrongiello BA, Corbett M, Lasenby J, Johnston N, McCourt M (2006). Factors influencing young children’s risk of unintentional injury: parenting style and strategies for teaching about home safety. J Appl Dev Psychol.

[20] Morrongiello BA, Widdifield R, Munroe K, Zdzieborski D (2014). Parents teaching young children home safety rules: implications for childhood injury risk. J Appl Dev Psychol.

[21] Morrongiello BA, McArthur BA, Bell M (2014). Managing children’s risk of injury in the home: does parental teaching about home safety reduce young children’s hazard interactions?. Accid Anal Prev.

[22] Gielen AC, Sleet D (2003). Application of behavior-change theories and methods to injury prevention. Epidemiol Rev.

[23] Ablewhite J, Peel I, McDaid L, Hawkins A, Goodenough T, Deave T (2015). Parental perceptions of barriers and facilitators to preventing child unintentional injuries within the home: a qualitative study. BMC Public Health.

[24] Kendrick D, Maula A, Stewart J, Clacy R, Coffey F, Cooper N (2012). Keeping children safe at home: protocol for three matched case–control studies of modifiable risk factors for falls. Inj Prev.

[25] Wynn P, Stewart J, Kumar A, Clacy R, Coffey F, Cooper N (2014). Keeping children safe at home: protocol for a case–control study of modifiable risk factors for scalds. Inj Prev.

[26] Majsak-Newman G, Benford P, Ablewhite J, Clacy R, Coffey F, Cooper N (2014). Keeping children safe at home: protocol for a matched case-control study of modifiable risk factors for poisoning. Inj Prev.

[27] Patton M (1990). Qualitative evaluation and research methods.

[28] Department for Communities and Local Government. English Indices of Deprivation 2010. Available at https://www.gov.uk/government/statistics/english-indices-of-deprivation-2010. [Accessed February 27, 2015]

[29] Smithson J, Garside R, Pearson M (2011). Barriers to, and facilitators of, the prevention of unintentional injury in children in the home: a systematic review and synthesis of qualitative research. Inj Prev.

[30] Ablewhite J, Peel I, McDaid L, Hawkins A, Goodenough T, Deave T, et al. Parental perceptions of barriers and facilitators to preventing child unintentional injuries within the home: a qualitative study. BMC Public Health. 2015; in press. 10.1186/s12889-015-1547-2 PMC4392794 25885179

[31] Bowling A (2002). Research methods in health.

